# Preconditioning boosts regenerative programmes in the adult zebrafish heart

**DOI:** 10.1098/rsob.160101

**Published:** 2016-07-20

**Authors:** Anne-Sophie de Preux Charles, Thomas Bise, Felix Baier, Pauline Sallin, Anna Jaźwińska

**Affiliations:** Department of Biology, University of Fribourg, Chemin du Musée 10, 1700 Fribourg, Switzerland

**Keywords:** myocardium, cardiomyocyte, non-mammalian animal model, heart regeneration, thoracotomy, zymosan

## Abstract

During preconditioning, exposure to a non-lethal harmful stimulus triggers a body-wide increase of survival and pro-regenerative programmes that enable the organism to better withstand the deleterious effects of subsequent injuries. This phenomenon has first been described in the mammalian heart, where it leads to a reduction of infarct size and limits the dysfunction of the injured organ. Despite its important clinical outcome, the actual mechanisms underlying preconditioning-induced cardioprotection remain unclear. Here, we describe two independent models of cardiac preconditioning in the adult zebrafish. As noxious stimuli, we used either a thoracotomy procedure or an induction of sterile inflammation by intraperitoneal injection of immunogenic particles. Similar to mammalian preconditioning, the zebrafish heart displayed increased expression of cardioprotective genes in response to these stimuli. As zebrafish cardiomyocytes have an endogenous proliferative capacity, preconditioning further elevated the re-entry into the cell cycle in the intact heart. This enhanced cycling activity led to a long-term modification of the myocardium architecture. Importantly, the protected phenotype brought beneficial effects for heart regeneration within one week after cryoinjury, such as a more effective cell-cycle reentry, enhanced reactivation of embryonic gene expression at the injury border, and improved cell survival shortly after injury. This study reveals that exposure to antecedent stimuli induces adaptive responses that render the fish more efficient in the activation of the regenerative programmes following heart damage. Our results open a new field of research by providing the adult zebrafish as a model system to study remote cardiac preconditioning.

## 1. Introduction

‘Was mich nicht umbringt, macht mich stärker’. (What does not kill me makes me stronger.) This statement from the German philosopher Friedrich Nietzsche (1844–1900) is a nearly prefect definition of the intriguing phenomenon referred to as preconditioning. It is more formally described as the induction of cellular survival and pro-regenerative programmes by transient exposure to non-lethal noxious stimuli, which increase the resistance of tissues to further harmful injuries. This physiological adaptation of tissues to subsequent damage was discovered three decades ago in the heart [1] but has in the meantime been described in a large variety of mammalian tissues, such as liver, kidney, skeletal muscle and nervous system [2,3]. Numerous studies performed on mammalian models of myocardial infarction have shown that subjecting hearts to ischaemic preconditioning before ventricular infarction led to a reduction of the myocardial infarct size and to a better recovery of cardiac function [4].

At first, a preconditioning effect was obtained by exposing heart to four brief episodes of coronary occlusion interspersed with reperfusion periods. The transposition of preconditioning into clinics has been possible thanks to the development of non-invasive cardiac preconditioning strategies using remote stimuli, such as the application of cycles of inflation–deflation using a blood pressure cuff placed on the upper arm [5,6]. Since then, multiple alternative triggers not based on ischaemia have been proposed, including peripheral nociception (topical application of capsaicin or surgical skin incision) and direct and transcutaneous nerve stimulation (electroacupuncture) [7]. These findings suggest that preconditioning-induced protective programmes can be activated in an inter-organ response to a wide range of transient aversive stimuli.

The mechanisms underlying the transport of the cardioprotective signal from the preconditioned tissue to the target organ remain unclear, but include both humoral and neural aspects [7,8]. Several studies have implicated a blood-borne factor as a mediator of cardioprotection. Indeed, the protected phenotype can be transferred from a preconditioned to a non-preconditioned individual via blood transfusion. This concept of a humoral signal transfer has been reproduced in several mammalian species, such as rabbit, rat and pigs [9–13]. Even though several factors, such as bradykinin, adenosine, SDF-1α or endocannabinoids, have been proposed, the identity of the humoral mediators of cardioprotection remains unclear [4,7,8]. The second aspect, namely the neural hypothesis, postulates that the preconditioned tissue produces factors that act locally to activate sensory afferent neurons and initiate the cardioprotective programmes [7,8]. In fact, nerve transection has been reported to abolish preconditioning-induced cardioprotection [14], while a direct stimulation of the sensory nerve reproduced the protected phenotype observed after preconditioning [15,16].

The protected phenotype after ischaemic preconditioning appears in two subsequent waves. The first one, the acute form, confers immediate cardioprotection, but its effects fade within 4 h. The second wave appears after 24 h, and lasts for several days. This delayed cardioprotection relies on the increased expression of protective proteins, while acute cardioprotection involves the recruitment of available signalling modules [17]. Preconditioning has raised an important clinical interest and has led to the publication of a large number of studies, identifying over 100 different signalling molecules and mechanisms. Acute cardioprotective pathways, which include NO/PKG, SAFE (STAT3/5 activation) or RISK pathway (PI3K, Akt, ERK activation), converge at the mitochondria, pointing to this organelle as a key effector of early cardioprotection [18]. The transcriptional upregulation of cardioprotective proteins observed during delayed cardioprotection is initiated by at least two signalling pathways that act in parallel: the PKCɛ/NF-κB and SAFE pathways [17,19].

Remarkably, preconditioning has also been reported in fish, such as rainbow trout and Atlantic cod, suggesting a common evolutionary origin of this phenomenon in vertebrates [20]. Over the last decade, the zebrafish has proved itself to be an extremely interesting model to study heart regeneration. In contrast to mammals, zebrafish cardiomyocytes (CMs) remain responsive to mitogenic signals throughout their life. This provides an efficient mechanism of regeneration after injury, leading to a full cardiac recovery within 30–60 days. Despite its high clinical interest, the cardioprotective mechanisms triggered by preconditioning have never been addressed using the zebrafish as a model system.

In this study, we described two independent models of cardiac preconditioning in the adult zebrafish. We used either a skin incision of the thorax (thoracotomy) or sterile inflammation in the peritoneum as the preconditioning stimulus. In contrast to mammalian models, preconditioning promoted the re-entry of cardiac cells into the cell cycle and led to a modification of the ventricular architecture. In addition, we observed an induction of more classical cardioprotective programmes in the epicardium shortly after the preconditioning stimulus. Remarkably, fish preconditioned a few days before heart injury exhibited an accelerated regeneration, illustrated by an increased rate of re-entry of CMs into the cell cycle, more CM dedifferentiation and better cell survival.

## 2. Material and methods

### 2.1. Animal procedures

This work was performed with fully grown adult fish at the age of 12–24 months. Wild-type fish were AB (Oregon), transgenic fish lines were *cmlc2:DsRed2-nuc* [21] and *cmlc2:EGFP* [22]. The control and preconditioned animals for each experiment were siblings that were maintained in the same density and food conditions. Before every procedure, fish were anaesthetized with tricaine (Sigma-Aldrich). To perform thoracotomy, fish were placed ventral side up on a damp sponge and a small incision was made through the thorax skin with iridectomy scissors. The peritoneal injections were performed by injection of 2 µl of solution into the abdomen of the fish using a glass microcapillary connected to the Femtojet transjector (Eppendorf). The lipopolysaccharides (LPS; Sigma-Aldrich) and Zymosan (Sigma-Aldrich) were injected at a concentration of 10 mg ml^−1^ in Hank's solution (20 µg of immunogenic particles injected per fish). Cryoinjuries were performed as described previously [23,24]. For bromodeoxyuridine (BrdU) incorporation experiments, the animals were maintained in 5 mg ml^−1^ BrdU (B5002; Sigma-Aldrich) for 7 or 30 days. During all treatments, fish were fed and solutions were changed every third day.

### 2.2. Immunohistochemistry and histology

At the end of each experiment, the hearts were collected and fixed overnight at 4°C in 2% paraformaldehyde. They were then rinsed in PBS and equilibrated in 30% sucrose before embedding in Tissue-Tek OCT compound (Sakura Finetek Europe B.V.) and cryo-sectioned at a thickness of 16 µm. The immunohistochemistry procedures were performed as previously described [23]. The following primary antibodies were used: rabbit anti-MCM5 at 1 : 5000 (kindly provided by Soojin Ryu, Heidelberg), mouse anti-p-Histone 3 at 1 : 200 (Millipore, Clone 3H10), rabbit anti-Mef2 at 1 : 100 (Santa Cruz Biotech SC-313), rabbit anti-DsRed (Clonetech, 632496) at 1 : 200 and rat anti-BrdU at 1 : 100 (Abcam, ab6326). To visualize the cardiac muscle, sections were incubated for 30 min with Phalloidin-Atto 647N (Sigma-Aldrich) at a dilution of 1 : 500. For the BrdU immunostaining, the slides were incubated in 2 M HCl in PBS with 0.3% Triton-X for 45 min before the immunohistochemistry procedure. The Alexa-Fluor-conjugated secondary antibodies (Jackson Immunoresearch) were used at 1 : 500, and DAPI was used at 1 : 2000. Haematoxylin and eosin (H & E) staining was performed as previously described [15].

### 2.3. TUNEL assay

For TUNEL reactions, the cryosections were postfixed for 10 min in 1% formalin, washed twice for 5 min in PBS and pretreated in precooled ethanol : acetic acid 2 : 1 for 5 min at −20°C. After washing in PBS, DNA breaks were elongated with Terminal Transferase (Roche) and Digoxigenin-dUTP solution (Roche) as described previously [25]. The reaction was stopped by incubation in 300 mM NaCl, 30 mM sodium citrate for 10 min, followed by washing in PBS. The staining with fluorescein conjugated anti-digoxigenin was performed according to the manufacturer's protocol (Roche).

### 2.4. Image analysis and quantification

After antibody staining, cardiac tissue imaging was performed at 20× magnification with confocal microscopes (Leica TCS-SP5 and Leica TCS-SPE-II). At least three different pictures were taken for each heart. Here, *n* represents the number of fish used in the experiment. ImageJ software was used to perform the subsequent image analysis. The number of proliferating non-CM nuclei was obtained by subtracting the number of *cmlc2:DsRed2-nuc*-positive nuclei from the total number of nuclei. To quantify the number of proliferating nuclei, the images of cell-cycle markers (MCM5, PH3, BrdU) were superimposed with the images of nuclei markers (DAPI, *cmlc2:DsRed2-nuc*, Mef2). The number of apoptotic nuclei was assessed by the superimposition of DAPI with the TUNEL labelling. To define the average myocardium thickness per section, we selected ventricles of a similar shape and size for control and preconditioning groups. We measured the thickness of the compact myocardium at four positions at the up, right, down and left side for each of the ventricular sections. The positions were selected at places where the compact and the trabecular myocardiums were clearly segregated and where the thickness of the compact myocardium was representative of the neighbouring positions. The average value of these measurements was then normalized to the maximal myocardial length. All results are expressed as the mean ± s.e. of the mean. Unless specified, *p*-values were obtained by performing the *t*-test.

### 2.5. *In situ* hybridization

Several digoxigenin-labelled RNA antisense probes were generated by PCR amplification of specific cardiac cDNA sequences. The forward (F) and reverse (R) primers are listed in table 1. The reverse primers were synthesized with the addition of T3 polymerase. After hybridization, the probes were detected by the use of anti-digoxigenin AP-conjugated antibody (Dig labeling system, Roche).

**Table 1.** List of the primers used for *in situ* hybridization and qRT-PCR.

application	gene	gene ID	forward primer 5′->3′	reverse primer 5′->3′	product length
qRT-PCR	*b-actin*	ENSDARG00000037870	TTGGCAATGAGAGGTTCAGG	TGGAGTTGAAGGTGGTCTCG	55 bp
	*hmox1a*	ENSDART00000030890	GCTCAGCTACCAGAAAGGACAG	CTGTCCAGCTCTTCCTCCAG	99 bp
	*hspa5*	ENSDART00000169404	TCTCCACTGCTTCCGACAAC	CAGATGGTTGTCTTTGGTCAGG	88 bp
*in situ*	*txn*	ENSDART00000064789	GCTCAAACGACACACGAGC	GTTTTCATTTCATACAAAGCCAACA	643 bp
	*cxcl12a*	ENSDART00000053946	AGTTCCTCCACACACCCAAC	AAACACGGAGCAAACAGGAC	452 bp

### 2.6. Quantitative real-time PCR

RNA was extracted according to the Trizol reagent manual (Life Technologies) with the use of MaXtract High Density tubes. cDNA was synthesized with the Super-Script-II Reverse-Transcriptase (Invitrogen) using 1.5 µg of RNA. The primers used for different amplifications are listed in table 1.

## 3. Results

### 3.1. Thoracotomy is sufficient to induce cardiomyocyte re-entry into the cell cycle

Previous studies in the zebrafish have shown that CM proliferation is strongly induced in surgically damaged heart after either amputation or cryoinjury [23,24,26,27]. In both injury models, the first procedure is an incision into the thoracic cavity to gain access to the heart [24]. Interestingly, our data showed that the initial surgical manoeuvre by itself is sufficient to stimulate the entry of CMs into the cell cycle, even when it was not followed by heart injury [23]. This suggests that the regenerative programme of zebrafish CMs could be markedly activated by a noxious stimulus. Accordingly, we hypothesized that thoracotomy may represent a preconditioning model, in which chest injury is sufficient to elicit an activation of the heart in the absence of any myocardial damage. Upon completion of the thoracic incision, the chest is not sewn but left for spontaneous healing. Remarkably, re-epithelialization of the wound took place within three days, while underlying connective tissue healed in approximately one week (electronic supplementary material, figure S1).

**Figure 1.**
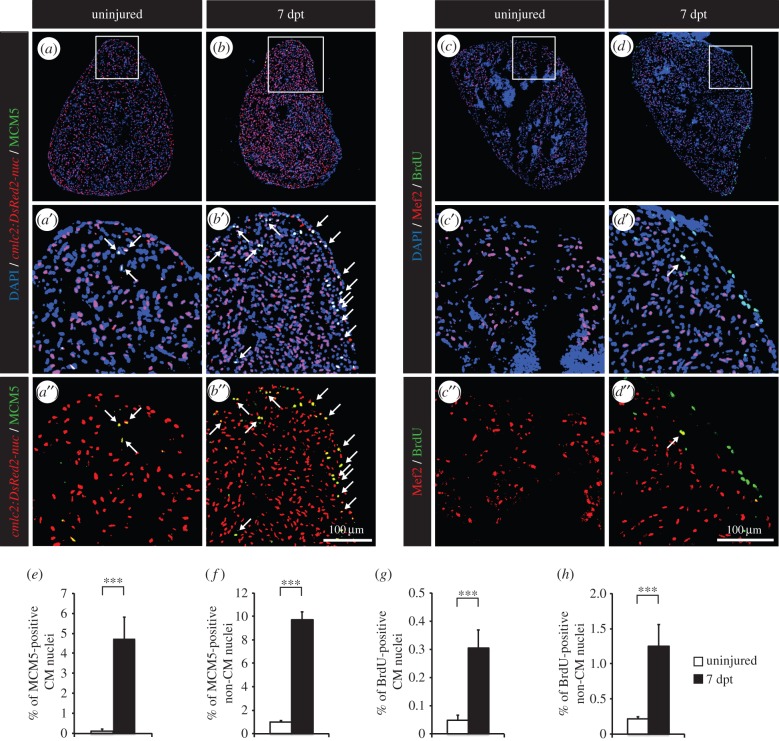
Thoracotomy triggers cell proliferation in the intact zebrafish heart. (*a,b*) Representative sections of hearts of transgenic fish *cmlc2:DsRed2-nuc* (red), which demarcate cardiomyocyte (CM) nuclei, labelled with the G1/S-phase marker MCM5 (green). (*c,d*) Representative sections of hearts after one week of BrdU (green) treatment. Mef2 staining (red) was performed to differentiate CM nuclei from non-CM nuclei. (*a*’,*b*’,*c*’,*d*’,*a*”,*b*”,*c*”,*d*”) Higher magnifications of the framed area shown in the images that are labelled with the same letter without the prime symbol. The same rule applies to all the subsequent figures. Arrows indicate double-positive nuclei. (*e*–*h*) Quantification of MCM5- and BrdU-positive CM and non-CM nuclei. (*n* ≥ 4 hearts; ≥2 sections per heart; ***p < 0.001).

First, we aimed to reproduce our initial observation that thoracotomy is sufficient by itself to induce the re-entry of CMs into the cell cycle. For this purpose, immunohistochemistry against minichromosome maintenance complex component 5 (MCM5), a marker of G1/S phase [28], was performed to quantify the entry of CMs into the cell cycle (figure 1*a*,*b*,*e*). To distinguish CM from non-CM nuclei, we used *cmlc2:DsRed2-nuc* fish, which express a nuclear form of DsRed under the control of the *cardiac myosin light chain 2* promoter. In control uninjured fully grown zebrafish, the ratio of MCM5-positive CMs ranges from 0.1 to 0.5%. Remarkably, at 7 days post-thoracotomy (dpt), about 5% of CMs were cycling. Proliferation of non-CM nuclei was similarly enhanced, with a 10-fold increase in MCM5-positive non-CM nuclei (figure 1*f*). To confirm these results, we used BrdU as a second cell-cycle marker. Fish were immersed in BrdU for 7 days following thoracotomy. Consistently, a sixfold increase in BrdU-positive CM and non-CM nuclei was detected at 7 dpt (figure 1*c*,*d*,*g*,*h*). Taken together, these observations indicate that the systemic reaction which is set in motion by the thoracotomy procedure elicits a global increase in the cell-cycling activity of the zebrafish heart.

**Figure 2.**
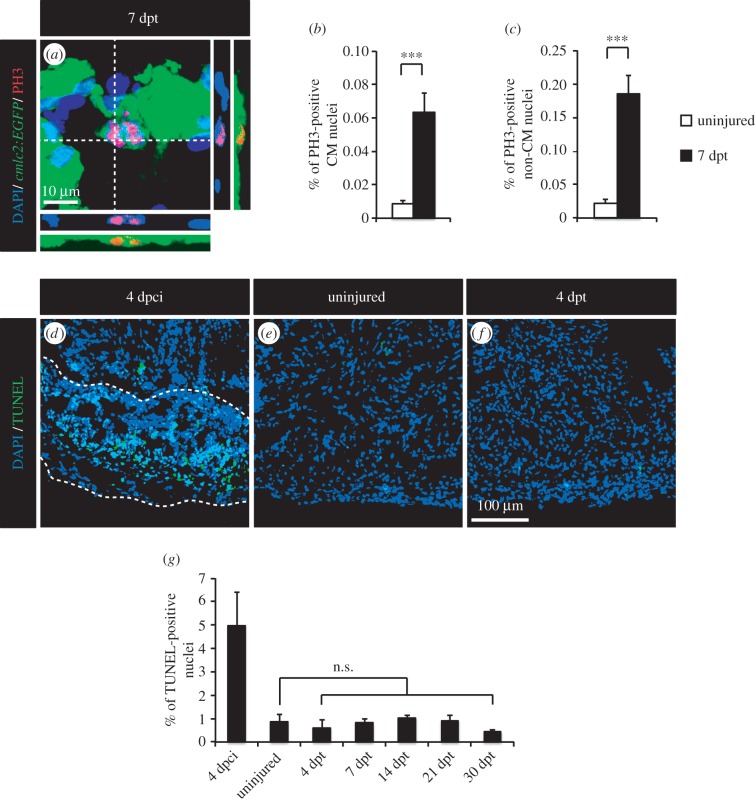
Thoracotomy stimulates mitotic events in the intact heart without apoptotic turnover of the newly generated cells. (*a*) Representative image of a mitotic CM in an intact heart of transgenic fish *cmlc2:EGFP* at 7 dpt. Orthogonal projections demonstrate a colocalization between PH3 (red), GFP (green) and DAPI (blue) staining. (*b,c*) Quantification of PH3-positive CM and non-CM nuclei at 7 dpt. Mef2 was used as a CM nuclei marker. (*d–f*) Representative images of the TUNEL assay (green) at 4 dpci (days post-cryoinjury; positive control for apoptosis with the post-infarcted area surrounded with a dashed line), in uninjured hearts and at 4 dpt. (*g*) Quantification of TUNEL-positive nuclei in hearts at 4 dpci, in uninjured hearts and at different time points post-thoracotomy (*n* ≥ 4 hearts; ≥2 sections per heart; n.s., non-significant; ***p < 0.001).

Then, the progression of the CM cell cycle was analysed. CMs can undergo DNA replication without completing their cell cycle. In humans, polyploidization occurs during postnatal growth and in response to myocardial stress [29]. To test whether the dividing CMs generated after thoracic incision entered mitosis, we used phospho-(Ser10)-histone H3 (PH3) as a marker of condensed chromosomes [30]. A sevenfold increase in the number of PH3-positive CM nuclei was observed at 7 dpt (figure 2*b*), while a ninefold increase was detected for non-CM nuclei (figure 2*c*). Clear examples of CM nuclear division were detected (figure 2*a*), indicating that thoracotomy leads to the formation of new CM nuclei.

To determine whether the enhanced CM mitotic activity was associated with dedifferentiation, we assessed the expression of embryonic isoform of cardiac myosin heavy chain (embCMHC), which has been shown to demarcate the margin of the remaining myocardium in the vicinity of the damaged tissue after amputation or cryoinjury [31]. The immunofluorescence analysis revealed that embCMHC was not induced after thoracotomy (data not shown). This result suggests that MCM5-positive CMs of the preconditioned hearts do not markedly revert to the immature state, which would facilitate cardiogenesis.

Next, we investigated whether the increased cycling activity observed in the absence of heart injury was followed by an elimination of supernumerary cells by apoptosis. TUNEL staining was performed at different time points after thoracic incision (4, 7, 14, 21 and 30 dpt), and at 4 days post-cryoinjury (dpci) as a positive control (figure 2*d*–*g*). Although an increased cell death was detected at 4 dpci, no difference was noticed between uninjured and thoracic-wounded animals, indicating that the induction of proliferation does not concur with an increased apoptosis and confirming that the thoracotomy procedure did not damage the cardiac tissue.

### 3.2. Thoracotomy induces architectural modifications of the myocardium

**Figure 3.**
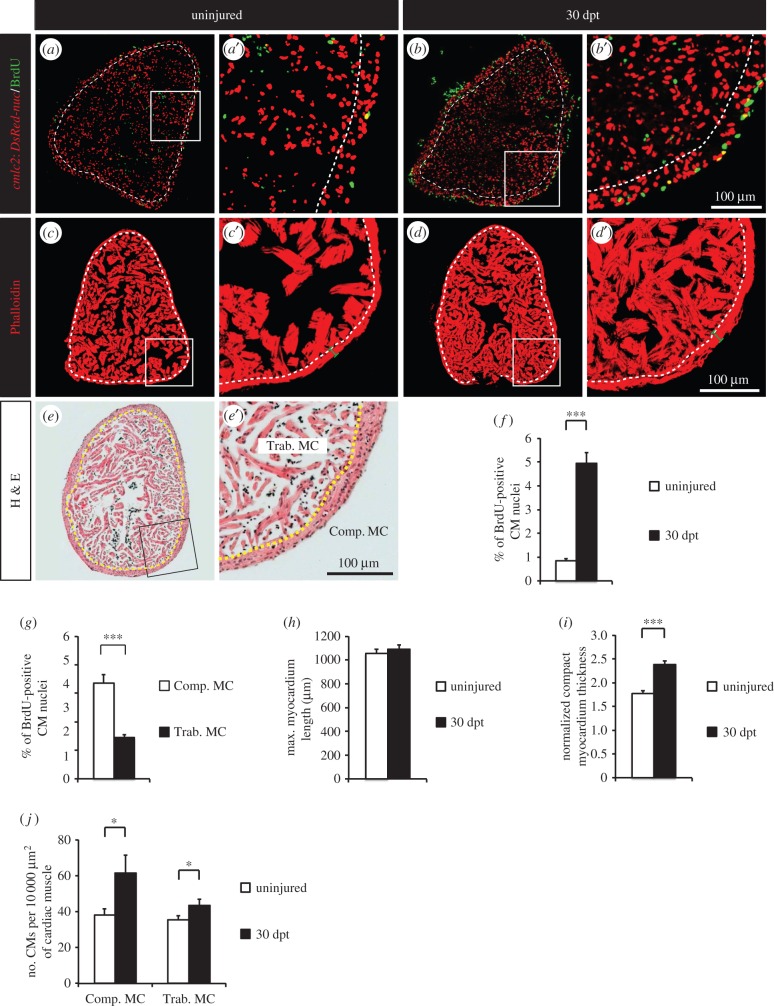
Thoracotomy triggers the remodelling of the heart architecture. (*a,b*) Uninjured and 30 dpt heart sections of transgenic zebrafish *cmlc2:DsRed2-nuc* (red) after one month of BrdU treatment (green). (*c,d*) Representative sections of hearts labelled with Phalloidin (red), a muscle marker, at 30 dpt. (*e*) Representative section of an uninjured heart labelled with haematoxylin/eosin (*h*,*e*) staining. Compact myocardium (Comp. MC) is composed of a dense layer of CMs, surrounding the trabecular myocardium (Trab. MC) with a spongy lumen. (*a*–*e*) Dashed lines separate compact and trabecular myocardium. (*f,g*) Percentage of BrdU-positive CMs in whole myocardium (*f*) or in the two distinct myocardium compartments (*g*). (*h*–*j*) Morphometric measurements of hearts at 30 dpt. No difference in heart size was noticed (*h*), but a thickening of the compact myocardium was observed (*i*). (*j*) The density of CMs (number of CMs per 10 000 µm^2^ of cardiac muscle) is higher at 30 dpt in comparison to uninjured control (*n* ≥ 4 hearts; ≥2 sections per heart; *p < 0.05; ***p < 0.001).

To test whether the proliferative response observed at 7 dpt was a lasting phenomenon, we immersed fish in BrdU for one month and assessed the profile of cycling CMs (figure 3*a*,*b*). We identified a fivefold increase in the number of BrdU-positive CMs at 30 dpt compared to 7 dpt (figure 3*f*). These results indicate that thoracotomy opens a long-term proliferative window in the zebrafish heart.

The zebrafish ventricle is composed of two distinct compartments: a dense cortical layer (compact myocardium) which surrounds a spongy inner structure (trabecular myocardium; figure 3*e*) [32]. We hypothesized that the proliferation induced after thoracotomy might not be evenly scattered through both myocardial compartments. To test this hypothesis, the spatial distribution of BrdU-positive CMs was characterized in fish treated with BrdU for 30 days. The CMs that entered the cell cycle during the month following thoracotomy were mainly located in the compact myocardium (figure 3*g*), confirming the result obtained at 7 dpt for MCM5-positive CMs (electronic supplementary material, figure S2).

Next, we asked if the observed increase in CM proliferation induced any long-term effect on the myocardium architecture. To this aim, we stained heart sections with Phalloidin, which binds to F-actin fibres present in muscle, and we quantified diverse morphometric parameters at 30 dpt (figure 3*c*,*d*,*h*–*j*). While the maximal heart length was unchanged (figure 3*h*), the thickness of the compact myocardium was increased at one month after thoracotomy (figure 3*i*). In addition, the number of CM nuclei per square micrometre was larger after thoracotomy (figure 3*j*). This increase was higher in the compact myocardium than in the trabecular compartment, supporting the notion that new CMs accumulate in the compact myocardium after thoracotomy and lead to a thickening of this outer myocardial compartment.

### 3.3. Intraperitoneal injection of immunogenic particles induced cardiomyocyte re-entry into the cell cycle

**Figure 4.**
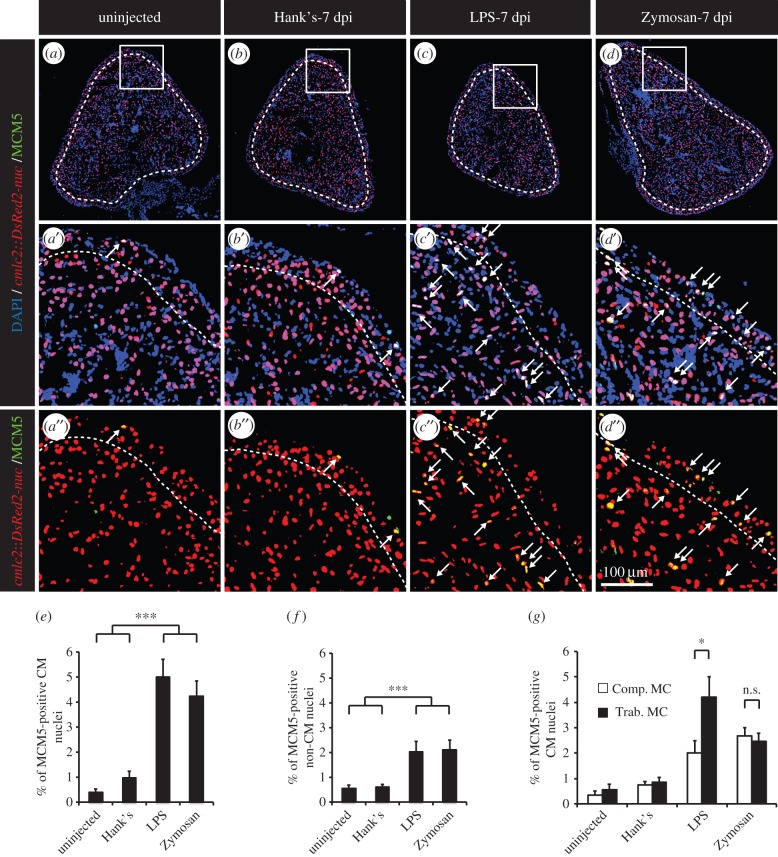
A single intraperitoneal injection of immunogenic particles triggers the reentry of CMs into the cell cycle. (*a*–*d*) Representative heart sections of uninjected transgenic *cmlc2:DsRed2-nuc* (red) fish controls (*a*) or injected with Hank's solution (*b*), LPS (*c*) or Zymosan (*d*) labelled with the cell-cycle marker MCM5 (green). Dashed lines separate compact and trabecular myocardium. Arrows indicate double-positive nuclei. (*e,f*) Quantification of MCM5-positive CM and non-CM nuclei. (*g*) Sublocalization of MCM5-positive CMs within the distinct myocardium compartment, the compact (Comp. MC) and trabecular (Trab. MC) myocardium (*n* ≥ 4 hearts; ≥2 sections per heart; n.s., non-significant; *p < 0.05; ***p < 0.001).

Even though no heart injury could be detected, we cannot exclude that the enhanced mitotic activity observed after thoracotomy might be triggered by a direct stimulation of the cardiac tissues by the osmotic or the mechanical stress created by the thoracic incision and not simply by the preconditioning stimulus. To test this possibility, we developed a second model of cardiac preconditioning using another remote stimulus. Specifically, an intraperitoneal (IP) inflammation was induced by a single injection of either immunogenic lipopolysaccharides (LPS) or Zymosan. LPS simulate a Gram-negative infection, whereas Zymosan mimics a fungal infection [33]. Outstandingly, at 7 days post injection (dpi), cell-cycle activity was enhanced in LPS- and Zymosan-injected fish, as compared to control fish injected with Hank's buffer (figure 4*a*–*f*), reaching levels similar to those observed after thoracotomy (figure 4*a*–*f*). Similar to the thoracotomy model, we assessed the spatial distribution of cycling CMs after the induction of a peritoneal sterile inflammation. In contrast to the thoracotomy model, MCM5-positive CMs were located throughout the whole heart of LPS- and Zymosan-injected fish (figure 4*f*).

### 3.4. Intraperitoneal injection of immunogenic particles stimulated thickening of the compact myocardium

**Figure 5.**
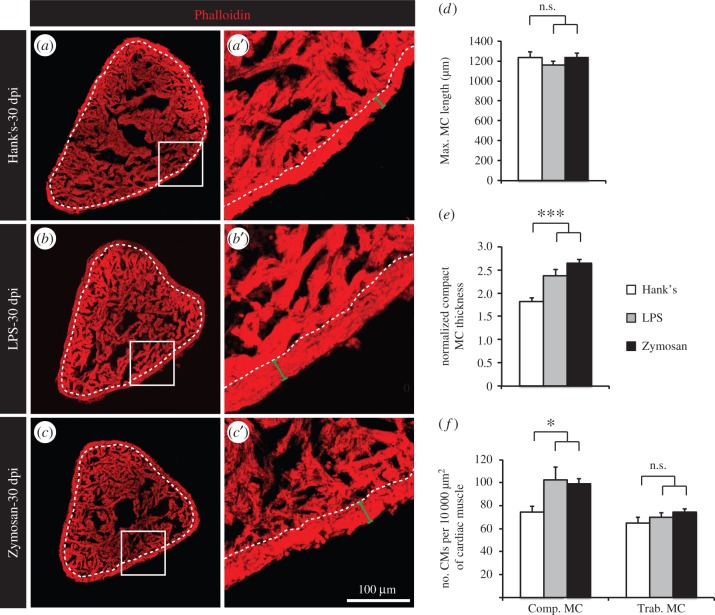
A single intraperitoneal injection of immunogenic particles induces a remodelling of the heart architecture. (*a*–*c*) Representative sections of hearts labelled with Phalloidin, a muscle marker, at 30 dpi. Dashed lines separate compact and trabecular myocardium. (*d*–*f*) Morphometric measurements of hearts at 30 dpi. No difference in heart size was noted (*d*), but a thickening of the compact myocardium was observed in fish injected with either LPS or Zymosan. (*f*) The density of CMs (number of CMs per 10 000 µm^2^ of cardiac muscle) was higher at 30 dpi in the compact myocardium of LPS- or Zymosan-injected fish (*n* ≥ 4 hearts; ≥2 sections per heart; n.s., non-significant; *p < 0.05; ***p < 0.001).

Then, we analysed the long-term effect of a single IP injection of either LPS or Zymosan on the myocardium architecture. Again, we stained heart sections with Phalloidin and quantified diverse morphometric parameters at 30 dpi (figure 5). Similar to thoracotomy, the maximal heart length was unchanged (figure 5*d*), but the thickness of the compact myocardium was increased (figure 5*e*). The analysis of *cmlc2:DsRed-nuc* transgenic fish revealed that a densification of CM nuclei in cardiac muscle was only detected in the compact myocardium at 30 dpt (figure 5*f*). In the trabecular myocardium, we observed that the muscle surface relative to the heart length (Phalloidin-positive area per maximal heart length) was increased in heart preconditioned with IP injection of LPS or Zymosan (data not shown). We did not notice the same phenomenon after thoracotomy (data not shown). This additional aspect of hearts preconditioned with a single IP injection of LPS or Zymosan indicates that architectural modifications might take place also in the trabecular myocardium in this model.

Taken together, our results show that similar phenotypic manifestations are observed after thoracotomy or IP injection of immunogenic particles, suggesting that both procedures trigger the activation of the similar molecular cascades.

### 3.5. Cardioprotective genes are expressed in the epicardium after thoracotomy

**Figure 6.**
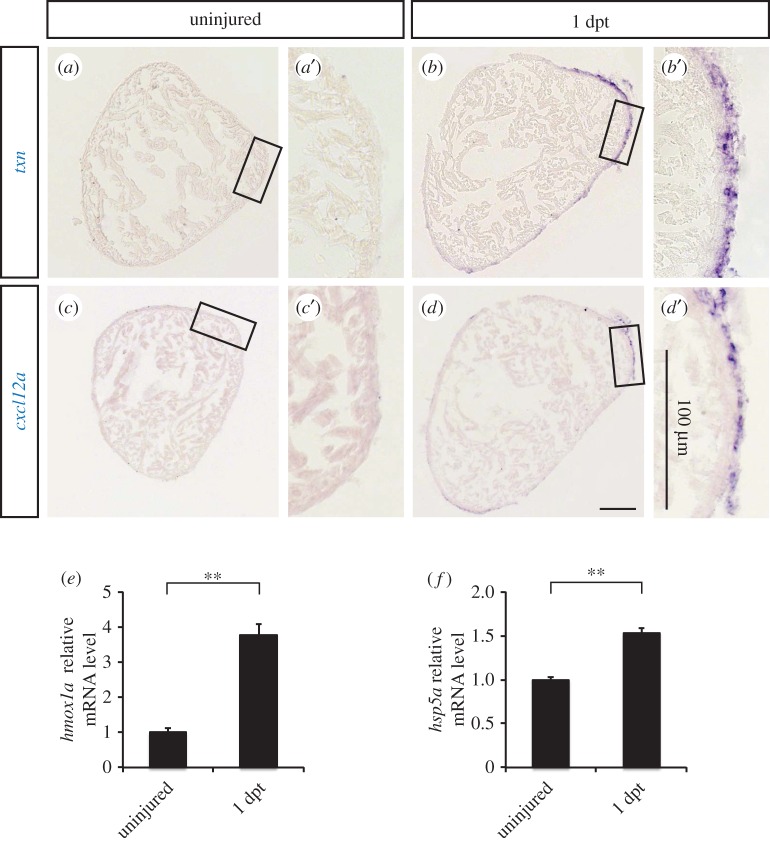
The expression of cardioprotective genes is induced in the epicardium after thoracotomy. (*a*–*d*) Representative images of *in situ* hybridization on heart sections with antisense *txn* and *cxcl12a* probes. (*e,f*) The relative expression level of *hmox1a* and *hpsa5* was tested by qRT-PCR (*n* ≥ 3; each replicate is a pool of six ventricles; **p < 0.01).

To determine whether thoracotomy induces phenotypes similar to those seen after ischaemic preconditioning, we tested the expression of several cardioprotective genes known to be induced by preconditioning in mammalian models. Thioredoxin plays a crucial role in the cellular defence against reactive oxygen species after ischaemic preconditioning [34], while CXCL12 (also known as SDF-1α) and its receptor CXCR4 are activated in response to preconditioning and promote cardiac protection against ischaemia/reperfusion damage [35]. The expression of their zebrafish orthologues (*txn*, *cxcl12a*, *cxcl12b*) was tested by *in situ* hybridization at 1 dpt. Interestingly, both *txn* and *cxcl12a* transcripts were upregulated in the epicardium at 1 day after the preconditioning stimulus (figure 6*a*–*d*). By contrast, we did not detect any change in the expression of *cxcl12b* (data not shown). Importantly, *txn* and *cxcl12a* were similarly upregulated in the epicardium after zymosan injection (data not shown). Next, we tested the expression of two heat-shock proteins induced after ischaemic preconditioning in rodents. The inability of *hmox1* knock-out mice to respond to preconditioning has highlighted the key role of this heat-shock protein in cardioprotection [36]. Other studies in rats have established an important role for HSPA5 (also known as GRP-78) in the protection of CMs against ATP depletion and oxidative stress after ischaemic preconditioning [37]. By qRT-PCR, we observed an increased expression of the zebrafish orthologues of these two heat-shock proteins, namely *hmox1a* and *hspa5*, shortly after the preconditioning stimulus at 1 dpt (figure 6*e*,*f*). Taken together, these results suggest that similar cardioprotective mechanisms are at stake in the adult zebrafish heart after thoracotomy and in mammalian ischaemic preconditioning models.

### 3.6. Subsequent preconditioning stimuli have additive effects

**Figure 7.**
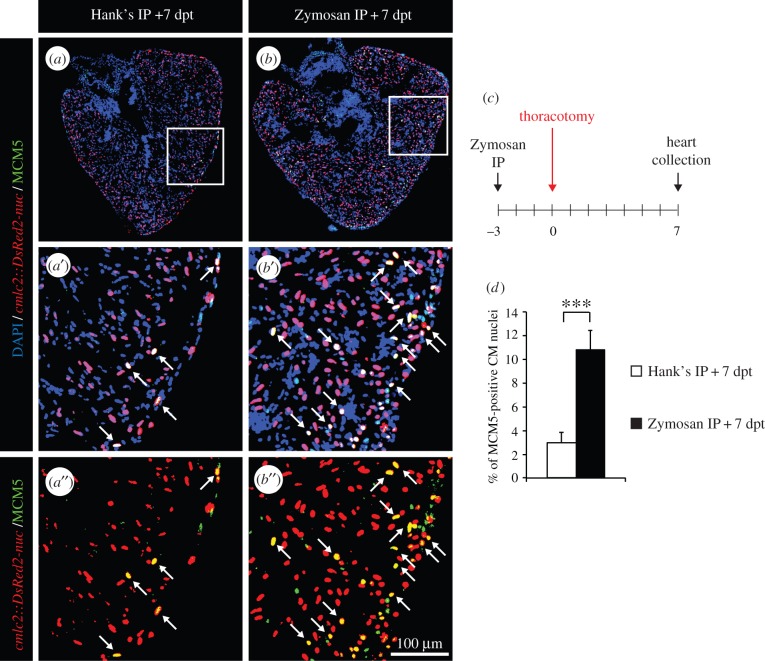
The effects on two subsequent preconditioning stimuli are additive. (*a,b*) Representative heart sections of transgenic *cmlc2:DsRed2-nuc* (red) fish labelled with the G1/S-phase marker MCM5 (green) at 7 dpt. Arrows indicate double-positive nuclei. (*c*) Experimental design. Fish were injected intraperitoneally (IP) with Zymosan 3 days before the thoracotomy procedure and cell-cycle activity was tested at 7 dpt. (*d*) Quantification of MCM5-positive CM nuclei at 7 dpt (*n* ≥ 4 hearts; ≥ 2 sections per heart; ***p < 0.001).

Next, we asked whether myocardial cell-cycle activity could be further boosted when two distinct preconditioning stimuli were combined. Accordingly, we performed an IP injection of Zymosan at 3 days before the thoracotomy procedure and, subsequently, assessed the rate of dividing CMs in the ventricle at 7 dpt (figure 7*c*). Remarkably, the percentage of MCM5-positive CMs increased by 3.6-fold when a single IP-injection of Zymosan was combined with thoracotomy, when compared with a control injection followed by thoracotomy (figure 7*a*,*b*,*d*). Our results demonstrate that two distinct non-lethal harmful stimuli have additive effects on the rate of cell-cycle re-entry of CMs and suggest that these procedures could be used as effective preconditioning stimuli before heart injury.

### 3.7. Using thoracotomy as a preconditioning stimulus in the adult zebrafish enhances regenerative programmes

To validate thoracotomy and IP injection of immunogenic particles as proper models of cardiac preconditioning in the zebrafish, we subjected fish to these non-lethal harmful stimuli a few days before wounding their hearts. In contrast to mammals, zebrafish are able to fully regenerate their heart following injury. Upon wounding, cardiac cells at the injury border reactivate developmental programmes, undergo mitosis and efficiently repopulate the damaged zone [23,26,27,38]. In addition, proliferative programmes are activated within the entire intact myocardium, where CMs distant from the wound enter the cell cycle without undergoing any obvious dedifferentiation [31].

To induce myocardial injury, we used the cryoinjury model, in which approximately 20% of the ventricle is damaged by exposure to a precooled metal probe [24]. Within the first week after the procedure, the initial inflammatory phase is tuned down and a collagenous matrix is deposited in the wounded area, providing a scaffold for the regeneration. By 7 days post-cryoinjury (dpci) the regenerative phase has been clearly initiated, and within four to eight weeks a complete restoration of the cardiac tissue can be observed [23,26,39].

**Figure 8.**
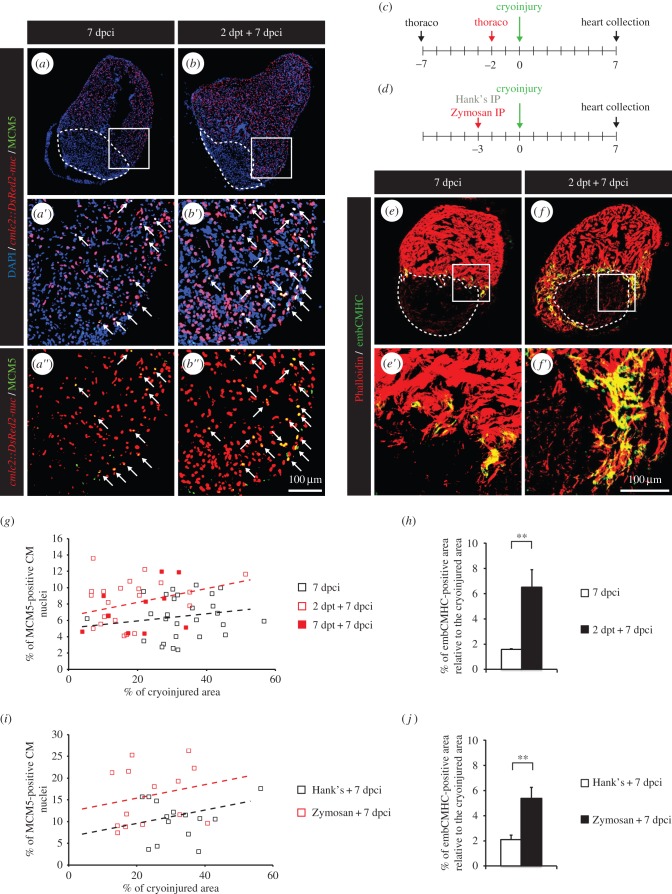
Heart regeneration is improved in fish subjected to thoracotomy a few days before the cryoinjury. (*a,b*) Representative sections of hearts of transgenic *cmlc2:DsRed2-nuc* (red) fish labelled with the G1/S-phase marker MCM5 (green) at 7 dpci. Arrows indicate double-positive nuclei. (*c,d*) Experimental design. (*c*) Fish were subjected to thoracotomy at 7 or 2 days before the cryoinjury procedure. (*d*) Fish were injected intraperitoneally (IP) with Zymosan at 3 days before the cryoinjury procedure. (*e,f*) Representative sections of hearts labelled with the muscle marker Phalloidin (red) and embCMHC (green), a marker of undifferentiated CMs. (*g,i*) Scatter plot representing the percentage of MCM5-positive CM nuclei as a function of the cryoinjured area while using thoracotomy (*g*) (grey squares = 7 dpci; red squares = 2 dpt + 7 dpci; black squares = 7 dpt + 7 dpci) or Zymosan-IP (*i*) (grey squares = Hank's + 7 dpci; black squares = Zymosan + 7 dpci) as preconditioning stimuli. (*h,j*) Quantification of the embCMHC-positive area after thoracotomy (*h*) or Zymosan-IP injection (*j*). A dashed line encircles the cryoinjured area (*n* ≥ 4 hearts; ≥2 sections per heart; **p < 0.01).

To maximize our chance to detect the effect of preconditioning on heart regeneration in the adult zebrafish, we focused our analysis at 7 dpci. At this time point, most regenerative programmes are active. We subjected fish to thoracotomy either 2 or 7 days before the cryoinjury procedure, referred to as a short-term preconditioning, and assessed CM proliferation in the ventricles at 7 dpci (figure 8*a*–*c*). The results were displayed by plotting the percentage of MCM5-positive CM nuclei as a function of the percentage of cryoinjured area for each heart section (figure 8*g*). We observed that the amount of dividing CMs was proportional to the size of the wound. Remarkably, CMs re-entered the cell cycle more efficiently in preconditioned hearts than in controls (figure 8*a*,*b*,*g*; electronic supplementary material, figure S3*b*). To confirm our observation of an enhancement of regeneration after preconditioning, we then examined the induction of developmental programmes in CMs in close vicinity to the wound, using embryonic cardiac myosin heavy chain (embCMHC) as a marker of undifferentiated CMs. In control unconditioned hearts, embCMHC staining can be detected starting from 4 dpci, it peaks between 7 and 10 dpci and decreases thereafter as regeneration progresses [31]. In preconditioned hearts, we observed a fourfold increase in the embCMHC staining at 7 dpci (figure 8*e*,*f*,*h*). Our data show a clear acceleration of the heart regeneration in fish preconditioned by thoracotomy a few days before the cryoinjury.

Interestingly, we could not observe a significant difference in the number of cycling CMs or in the size of embCMHC-positive myocardium between hearts preconditioned at 2 or 7 days before cryoinjury (figure 8*g*; data not shown). These results suggest that preconditioned hearts are able to retain their protected phenotype for at least one week. To test whether preconditioned hearts hold these cardioprotective capacities in the long term, we performed a thoracotomy 30 days before the cryoinjury procedure (electronic supplementary material, figure S4*a*). This long-term preconditioning did not lead to improvement of the cardiac regeneration as convincingly as that seen when thoracotomy was performed within the week preceding the cryoinjury (electronic supplementary material, figure S4*d*). The rate of cycling CMs as a function of the size of the wounded area was not higher in preconditioned fish (electronic supplementary material, figure S4*b*, S4*e*). Furthermore, no difference between control and preconditioned fish could be observed in the quantity of undifferentiated CMs (electronic supplementary material, figure S4*c*).

### 3.8. Using Zymosan intraperitoneal injection as a preconditioning stimulus in the adult zebrafish enhances regenerative programmes

To test whether IP injection of immunogenic particles could also be used to confer cardioprotection before cryoinjury, similar to thoracotomy, we performed a single IP injection of Zymosan at 3 days before the cryoinjury procedure (figure 8*d*). Again, CMs re-entered the cell cycle more efficiently in preconditioned hearts (figure 8*i*; electronic supplementary material, figure S3*d*). The initiation of developmental programmes in CMs at the injury border was stronger after Zymosan injection, as illustrated by a 2.5-fold increase in injury-normalized embCMHC staining (figure 8*j*). These results also establish IP injection of Zymosan as a valid preconditioning stimulus in the adult zebrafish.

### 3.9. Preconditioning in the adult zebrafish enhances survival programmes

**Figure 9.**
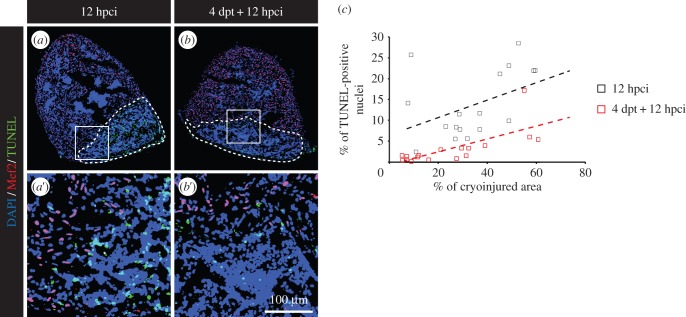
Preconditioning enhances cell survival after cryoinjury. (*a,b*) Representative pictures of the TUNEL assay (green) at 12 hpci in uninjured (*a*) or preconditioned fish (*b*). Mef2 labelling (red nuclei) is absent from the cryoinjured area. A dashed line encircles the wound. (*c*) Scatter plot representing the % of TUNEL-positive nuclei as a function of the cryoinjured area while using thoracotomy (black squares = 12 hpci; red squares = 4 dpt + 12 hpci) as preconditioning stimulus.

We noticed that the size of the cryoinjuries tended to be smaller after short-term preconditioning. This observation was mostly striking in our thoracotomy model (figure 8*g*), where the average cryoinjury covered 36.6 ± 2.6% of the control heart and only 20 ± 2.4% of the preconditioned hearts (electronic supplementary material, figure S3*a*). A milder phenotype was seen after Zymosan injection (figure 8*i*; electronic supplementary material, figure S3*c*). With this procedure, the injured areas represented on average 33 ± 2.4% of the surface of the heart in Hank's-injected fish and 24.4 ± 2.4% in Zymosan-injected specimens. In accordance with our previous results, we found no difference in the size of cryoinjuries after long-term preconditioning (electronic supplementary material, figure S4*d*). Based on these results, we asked whether preconditioning could promote survival programmes after cryoinjury. For this aim, TUNEL assay was performed at 12 h post-cryoinjury (hpci) in the hearts of control and preconditioned fish (figure 9*a*,*b*). Apoptosis was impressively decreased in preconditioned hearts (figure 9*c*), suggesting that preconditioning efficiently promote survival within a few hours after injury.

## 4. Discussion

Preconditioning can be considered as an evolutionary adaptation of organisms to activate protective/pro-regenerative programmes upon exposure to mild pathologic perturbations in order to better withstand the deleterious effects of ensuing harsh injuries. In the mammalian heart, short bouts of non-lethal cardiac or non-cardiac insults elicit cytoprotection against subsequent prolonged ischaemia and reperfusion [7]. The preconditioned phenotype is characterized by the elevated expression of pro-angiogenic, antioxidant and cardioprotective genes [17,19,40]. A mechanistic rationale for new drug therapies that mimic these powerful protective responses remains unfortunately still unclear.

In this study, we identified that heart preconditioning also occurs in the adult zebrafish. We described two independent models of remote preconditioning, namely thoracotomy and induction of a sterile IP inflammation. In our first model, a chest incision without heart injury was sufficient to invoke a proliferative phenotype in CMs and non-CMs in the ventricle during the time window of two to three weeks after the procedure. Thus, cardiac remodelling is not restricted to myocytes, but is a general feature of cells that build the fish heart. Remarkably, the CM cell-cycle re-entry as assessed by MCM5 expression was nearly as high as after cryoinjury [23]. However, as opposed to the regenerative process, we did not observe CM dedifferentiation in the preconditioned heart. It is possible that the rate of cardiogenesis is different between mature and immature proliferating CMs in preconditioning and regenerative processes, respectively. Moreover, the enhanced mitotic activity was mainly located in the compact myocardium and led to a thickening of this dense cortical myocardial layer one month after the initial preconditioning stimulus. This architectural alteration can increase the robustness of the heart. It is reasonable to assume that this phenotype is adaptive and renders the organ more resistant to a cardiac muscle injury. Indeed, several studies have identified a leading role of a compact myocardium during heart regeneration after ventricular resection [27,41,42]. Additional benefit of the more robust outer wall would be a limitation of the heart dysfunction immediately after ventricle damage, which enables the fish to better cope with damage. Certainly, the significance of an enlarged compact myocardial layer after thoracotomy requires further investigation. In general, the elucidation of the cytoprotective processes in the zebrafish model may uncover the mechanistic underpinnings for this clinically relevant phenomenon.

Remarkably, we observed a similar phenotype in our second model of cardiac preconditioning. A single IP injection of immunogenic particles led to an increased mitotic activity and to a long-term modification of the heart architecture. After thoracotomy, cycling cells were mainly detected in the compact myocardium, whereas they were scattered throughout the heart after LPS or Zymosan injection. The different localization of cycling cells in the ventricle after thoracic surgery and after the induction of an IP inflammation might result from the spatial pattern of stimuli used. Indeed, in thoracic-wounded fish, the outer surface of the heart is mainly in contact with the aversive stimuli. In fish injected with immunogenic particles, a systemic response appears, which progressively reaches the heart, probably via blood circulation. In this context, the whole myocardium is in contact with the aggressive components. Remarkably, LPS/Zymosan injections activated CM proliferation more selectively than thoracotomy. These finding should be further investigated in additional studies. Despite these variations in the profile of cycling CMs, both models exhibited a thicker compact myocardium, indicating architectural modification of the heart at one month after the preconditioning. The striking resemblance between both models suggests that these two remote preconditioning stimuli trigger the same molecular cascades in the target organ.

Although the mammalian heart studies are predominantly based on mild antecedent ischaemia/reperfusion, remote cardiac preconditioning has also been observed [8]. Similar to thoracotomy, skin incision models as conditioning stimuli have been described in mouse and dog [43,44]. In these models, preconditioning is dependent on skin nociception and on the activation of cardiac sensory and sympathetic nerves. As mammalian adult CMs are poorly responsive to mitogenic signals, the downstream effectors of preconditioning do not involve cell division pathways [19,45]. By contrast, cardiac preconditioning in the zebrafish promoted a hyperplastic growth of the heart. This striking difference between mammalian and teleost models of cardiac preconditioning is most likely due to the remarkable capacities of the zebrafish heart to respond to mitogenic signals throughout ontogenic life. Moreover, our results obtained in the adult zebrafish preconditioned heart are reminiscent of the observations made in mammalian preconditioned kidney or liver, where proliferation can be observed after ischaemic preconditioning [46–48]. In contrast to the adult mammalian heart, these adult tissues are still responsive to mitogenic signals [49,50]. Taken together, these observations emphasize the re-entry into the cell cycle as a classic protective reaction in tissues sensitive to mitogens.

In parallel to an increased cell-cycle activity, we observed an enhanced expression of more classical cardioprotective genes. We tested the expression of four zebrafish orthologues of mammalian genes activated after ischaemic preconditioning, namely *txn*, *cxcl12a*, *hmox1a* and *hspa5* [34–37]. All of them were increased 24 h after thoracotomy, suggesting that preconditioning elicits conserved cardioprotective mechanisms through species. Interestingly, our *in situ* hybridization revealed that *txn* and *cxcl12a* expression was restricted to the epicardium. This expression pattern highlights this cardiac compartment as a key mediator of cardioprotection and is consistent with a previous report that has demonstrated that the opening of the pericardial sac is sufficient to induce the expression of *raldh2*, a marker of the activated epicardium [51]. Thus, the epicardium is a dynamic tissue responsive to changes within the extra-cardiac space. Here, we have demonstrated that the stimulation of this sensitive mesothelial layer coincides with the activation of the CM cell cycle. Even though the capital role played by the epicardium in cardiac regeneration has already been examined in the zebrafish [52–54], further studies are needed to outline its function in the formation of a protected phenotype after preconditioning.

In mammalian models, preconditioning hearts before prolonged ischaemia reduces significantly the resultant infarct size. In contrast to mammals, the zebrafish heart heals naturally after injury within 30–60 days. In an organism in which regeneration is a highly efficient process, one can only expect a modest increase in the speed of recovery. To maximize our chances to see how preconditioning impacts on heart regeneration, we focused our analysis at 7 dpci, when most healing programmes are already induced. Remarkably, we observed a clearly enhanced regeneration in preconditioned hearts at this time point, independently of the used stimuli. The re-entry of CMs into the cell cycle was more efficient and more CMs exhibited hallmarks of dedifferentiation at the injury border. Moreover, we observed less apoptosis in the damaged area of preconditioned hearts shortly after the cryoinjury procedure. We also tried to assess regenerative scores at 30 dpci. Unfortunately, we did not detect a significant improvement of the regenerative status of preconditioned heart versus control at this late stage of cardiac healing. This disappointing outcome might be explained by the high inter-individual variability present during heart regeneration. At 30 dpci, the injured area of control non-preconditioned fish is sometimes almost completely resorbed, but can also still be visible [55,56]. Indeed, the speed of heart regeneration will depend on a large variety of parameters such as the initial size of cryoinjury, the level of stress or the general health status of the fish [56].

Outstandingly, the same cardioprotective capacities were observed in hearts preconditioned at 2 or 7 days before the cryoinjury procedure. By contrast, the protected phenotype was lost when a gap of one month was used between the thoracotomy and the cryoinjury. Taken together, our data indicated that preconditioned hearts are able to retain a stable cardioprotective phenotype for at least one week, regardless of the number of days between the preconditioning and the heart injury.

In summary, we proposed two independent models to study cardiac preconditioning using the adult zebrafish as a model system. In contrast to mammalian models, the increased expression of cardioprotective genes is accompanied by an increased mitotic activity leading to the long-term remodelling of the adult zebrafish myocardium architecture. The demonstration of the cardioprotective effects after heart injury of thoracotomy and IP inflammation validated these procedures as appropriate models for cardiac preconditioning in the zebrafish. Our findings open a new field of research that uses the zebrafish to study the cardiac preconditioning phenomenon. The understanding of how noxious stimuli can invoke subsequent intrinsic cell protective programmes to increase the global cardiac muscle tolerance creates an interesting perspective, which can promote new therapeutic strategies in prevention of ischaemic muscle diseases.

## Supplementary Material

Figure S1: Thoracic wound closes spontaneously within 3-4 days after thoracotomy.

## Supplementary Material

Figure S2: Sublocalization of cycling CMs at 7 dpt.

## Supplementary Material

Figure S3: Quantification of the cryoinjury size and of the number of MCM5-positive CM nuclei after cryoinjury.

## Supplementary Material

Figure S4: The cardioprotective phenotype is lost at 30 dpt.

## Supplementary Material

Table S1:List of the primers used for in situ hybridization and qRT-PCR
