# Supplementary material for: Preconditioning boosts regenerative programmes in the adult zebrafish heart
Source: Open Biol. 2016 Jul 20;6(7):160101. doi: 10.1098/rsob.160101 (PMC4967829; doi:10.1098/rsob.160101)
Supplement: Figure S1: Thoracic wound closes spontaneously within 3-4 days after thoracotomy. [file rsob160101supp1.pdf]

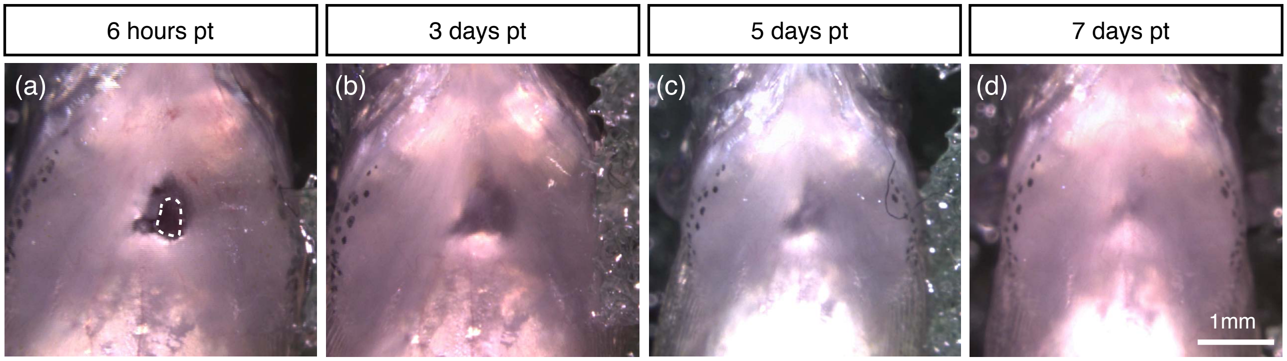

Figure S1: Thoracic wound closes spontaneously within 3-4 days after thoracotomy.

Photographs of the ventral side of the same fish at different time points after performing 1 mm-long incision through the body wall. (a) At 6 hours post thoracotomy (hpt), the thorax has an open wound seen as a dark window through the whitish dermis. A transparent epithelium already starts to cover the wound, reducing the diameter of the hole (encircled area). (b) At 3 days post thoracotomy (dpt), the wound has been completely sealed by the sheet of epidermis. (c) At 5 dpt, dermis has contracted around the wound. (d) At 7 dpt, the thoracic wound has been almost perfectly healed by the restoration of the dermal tissue.
