# Supplementary material for: Preconditioning boosts regenerative programmes in the adult zebrafish heart
Source: Open Biol. 2016 Jul 20;6(7):160101. doi: 10.1098/rsob.160101 (PMC4967829; doi:10.1098/rsob.160101)
Supplement: Figure S2: Sublocalization of cycling CMs at 7 dpt. [file rsob160101supp2.pdf]

(a)

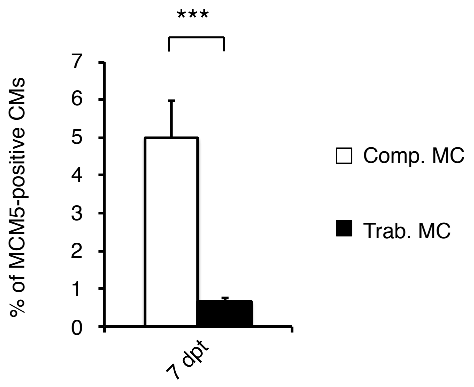

Figure S2: Sublocalization of cycling CMs at 7 dpt

(a) MCM5-positive CMs are mainly located in the compact myocardium at 7 dpt. ($n \geq 3$; ***$P < 0.001$).
