# Supplementary material for: Preconditioning boosts regenerative programmes in the adult zebrafish heart
Source: Open Biol. 2016 Jul 20;6(7):160101. doi: 10.1098/rsob.160101 (PMC4967829; doi:10.1098/rsob.160101)
Supplement: Figure S3: Quantification of the cryoinjury size and of the number of MCM5-positive CM nuclei after cryoinjury. [file rsob160101supp3.pdf]

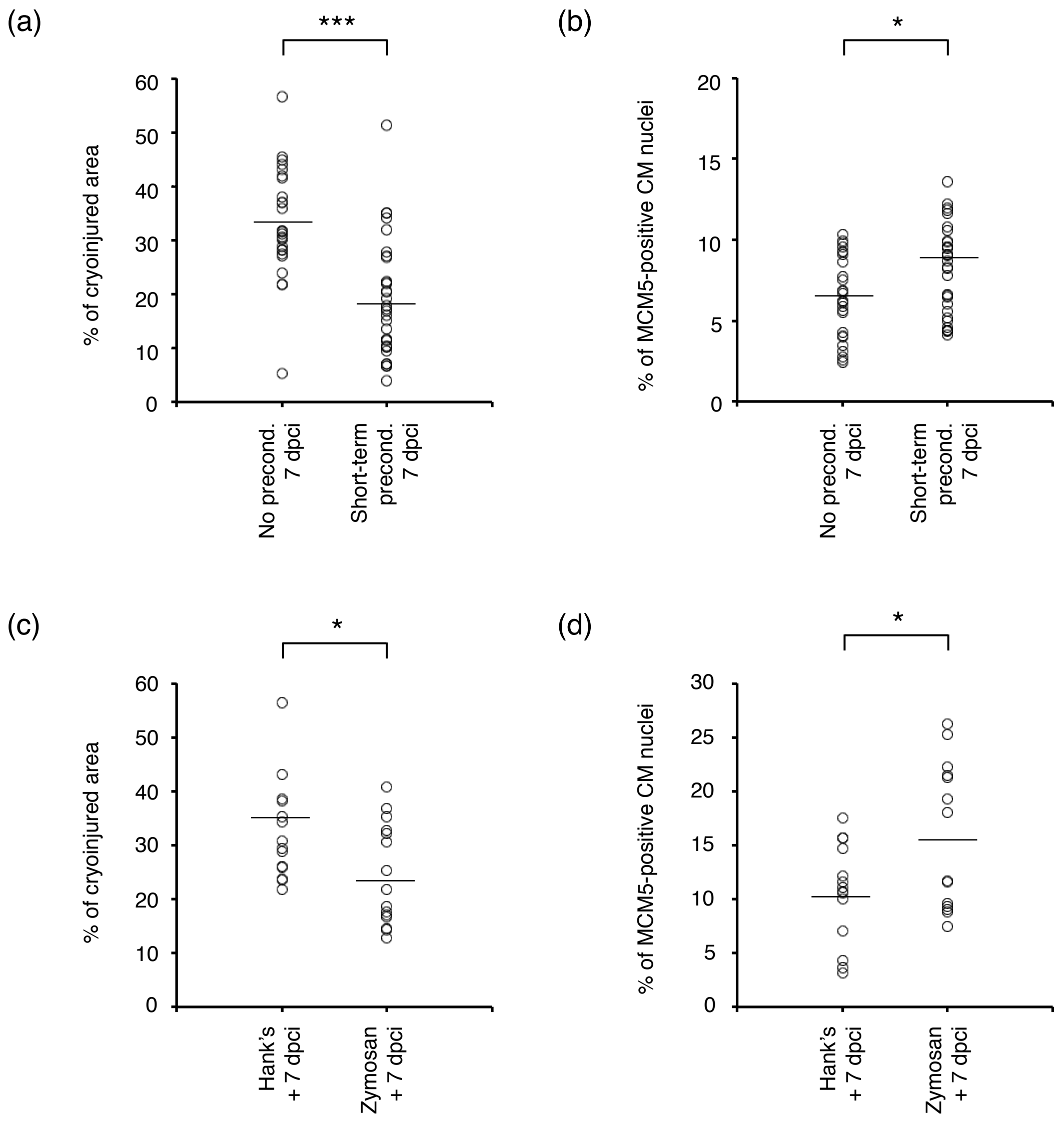

Figure S3: Quantification of the cryoinjury size and of the number of MCM5-postive CM nuclei after cryoinjury

(a-d) Quantification of the percentage of the croyinjured area and of MCM5-postive CM nuclei after short-term preconditioning using thoracotomy (a-b) or IP zymosan-injection (c-d) as a preconditioning stimuli. ($n \geq 4$ hearts; ≥ 2 sections per heart; *$P < 0.05$; ***$P < 0.001$).
