# Supplementary material for: Preconditioning boosts regenerative programmes in the adult zebrafish heart
Source: Open Biol. 2016 Jul 20;6(7):160101. doi: 10.1098/rsob.160101 (PMC4967829; doi:10.1098/rsob.160101)
Supplement: Figure S4: The cardioprotective phenotype is lost at 30 dpt. [file rsob160101supp4.pdf]

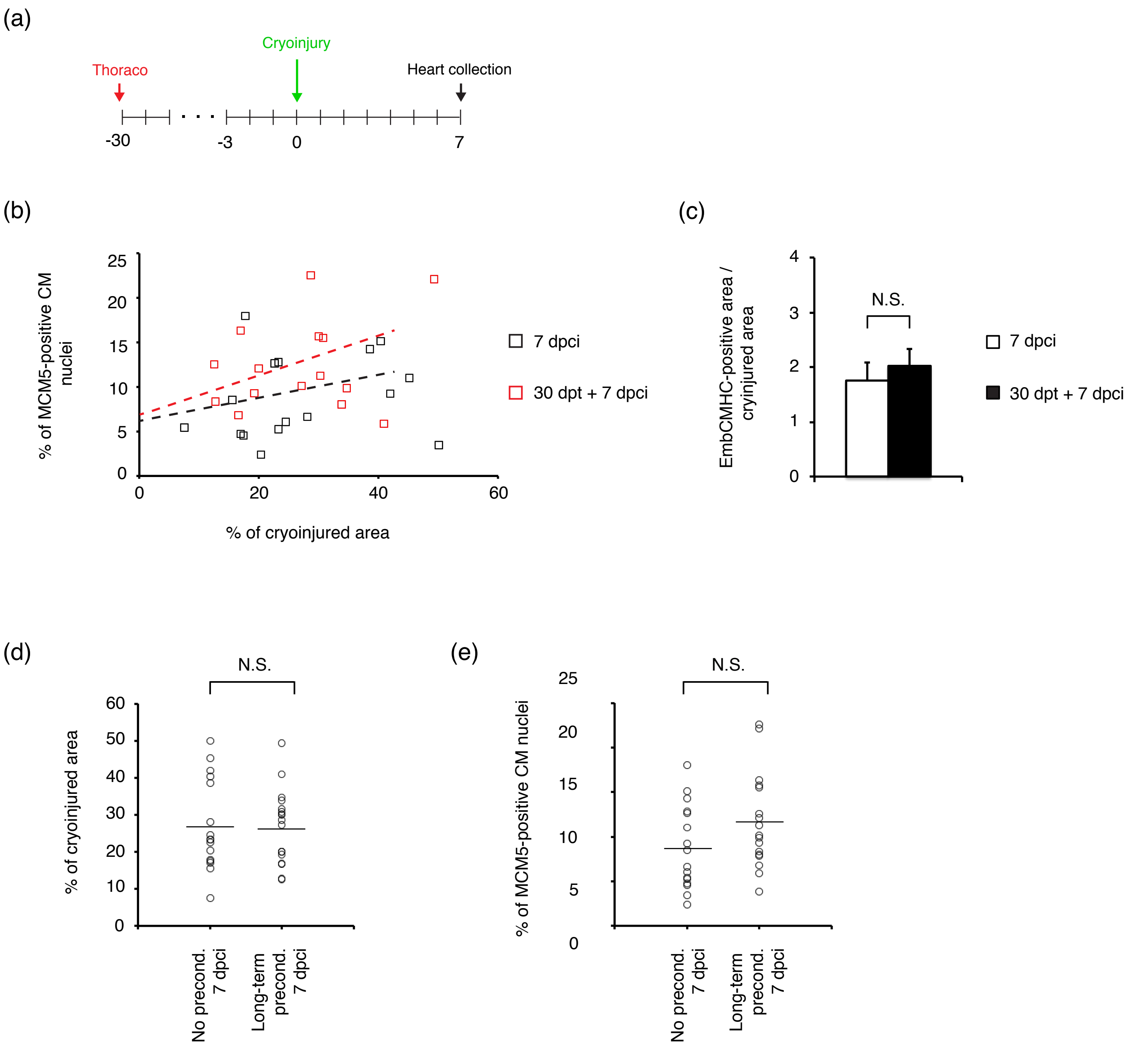

Figure S3: The cardioprotective phenotype is lost at 30 dpt.

(a) Experimental design. Fish were subjected to thoracotomy 30 days before the cryoinjury. (b) Scatter plot representing the % of MCM5-positive CM nuclei in function of the cryoinjured area while using thoracotomy (grey squares = 7 dpci; black squares = 30 dpt + 7dpci) as preconditioning stimulus. (c) Quantification of the EmbCMHC-positive area at 7 dpci. (d-e) Quantification of the percentage of the cryoinjured area and of MCM5-postive CM nuclei after long-term preconditioning uing thoracotomy as a preconditioning stimulus. (n ≥ 5)
